# Pathology and Molecular Diagnosis of Marek's Disease Virus in Chickens in Nigeria

**DOI:** 10.1155/sci5/5848535

**Published:** 2025-11-14

**Authors:** Olusegun Adesina Fagbohun, Aiki-Raji Comfort Oluladun, Theophilus Aghogho Jarikre, Olugbenga Olayinka Alaka, Ademola Adetokunbo Oyagbemi, Rofiat Damilola Adesina, Oluwaseun Olanrewaju Esan, Olumide Odunayo Akinniyi, Moses Olusola Adetona, Temidayo Olutayo Omobowale, Ufuoma Joan Mamoh, Ishmael Festus Jaja

**Affiliations:** ^1^Department of Veterinary Microbiology, Faculty of Veterinary Medicine, University of Ibadan, Ibadan, Oyo State, Nigeria; ^2^Department of Veterinary Pathology, Faculty of Veterinary Medicine, University of Ibadan, Ibadan, Oyo State, Nigeria; ^3^Department of Veterinary Physiology and Biochemistry, Faculty of Veterinary Medicine, University of Ibadan, Ibadan, Oyo State, Nigeria; ^4^Department of Veterinary Medicine, Faculty of Veterinary Medicine, University of Ibadan, Ibadan, Oyo State, Nigeria; ^5^Department of Anatomy, Faculty of Basic Medical Sciences, College of Medicine, University of Ibadan, Ibadan, Oyo State, Nigeria; ^6^Department of Livestock and Pasture Science, Faculty of Science and Agriculture, University of Fort Hare, Alice 5700, South Africa; ^7^Department of Agriculture and Animal Health, University of South Africa, Roodepoort, Johannesburg 1709, South Africa

## Abstract

Marek's disease caused by Marek's disease virus (MDV) serotype 1 is an economically important neoplastic disease of poultry. Diagnosis of this disease is usually based on clinical signs, postmortem lesions, and diagnostic tests like cytology, histopathology, and molecular-based methods. However, there might be the problem of inaccurate diagnosis in Nigeria. Employment of gross pathology, histopathology, immunohistochemistry, and polymerase chain reaction (PCR) coupled with sequence analysis provides a reliable approach to arrive at precise confirmatory diagnosis of the disease. Therefore, visceral organs including liver, proventriculus intestine, spleen, and heart samples were collected at postmortem examinations from two pullets suspected of having Marek's disease. Histopathology and immunohistochemistry, PCR amplification of a 576 bp fragment of the MDV glycoprotein L (gL) gene, and sequence analysis were employed in this diagnostic approach. Histopathological examinations of the liver, heart and proventriculus showed neoplastic pleomorphic cellular infiltration comprising lymphoblasts, lymphocytes, macrophages, and heterophils which correspond with the pathology of Marek's disease. Liver samples were positive for the virus using PCR. Sequence analysis based on phylogenetic tree reconstruction revealed the positive MDV. MDV sequences from this study clustered with MDV serotype 1 sequences retrieved from the GenBank. This approach provides a reliable and precise diagnosis of Marek's disease in chickens which is applicable to other avian diseases.

## 1. Introduction

The poultry industry in Nigeria is a very profitable venture that can contribute significantly to the country's economy [1]. However, the progress of this industry is being encumbered by infectious diseases, most importantly neoplastic diseases such as Marek's disease. The three most economically important neoplastic diseases of poultry are Marek's disease, avian leucosis, and reticuloendotheliosis [2]. Marek's disease is caused by Marek's disease virus (MDV) serotype 1 (Gallid herpesvirus 2) belonging to the family Herpesviridae, subfamily Alphaherpesvirinae and the genus Mardivirus [3]. The genus Mardivirus consists of other species such as Gallid herpesvirus 3 (GaHV-3), also known as MDV serotype 2, Meleagrid herpesvirus 1 (MeHV-1), also known as herpesvirus of turkey (HVT), Anatid herpesvirus 1, and the Columbid herpesvirus 1. MDV serotype 2 and HVT infect domestic fowls like MDV, but are apathogenic and nononcogenic [4]. The aforementioned avian tumor inducing viruses appear to have multipotent characteristics, as they can sometimes induce a variety of neoplasms of which present a diagnostic problem. This is because certain strains of these viruses induce some pathologic lesions that are difficult to distinguish from those induced by other unrelated viruses [5, 6]. Infiltration of diffuse lymphomas is seen in the liver and other visceral organs resembling Marek's disease. However, the pp38 antigen of MDV, occasionally in Marek's disease lymphomas is absent in avian leucosis and reticuloendotheliosis as well as the presence of MHC class II (Ia) antigens on Marek's disease lymphoma cells but are absent on reticuloendotheliosis cells [5, 7].

Furthermore, Marek's disease is characterized by pleomorphic cellular infiltrates in peripheral nerves, various organs and tissues including iris and skin, with immunosuppression phases [4] whereas it is uniform mononuclear cellular infiltrates in leucosis. Chicks are susceptible to horizontal transmission; the presence of immunity conferred by the maternal antibodies gives a degree of resistance to the disease [8]. However, most Marek's disease diagnoses in Nigeria are based on clinical and postmortem observations. More so, MDV field pathotypes are yet to be determined for their virulence, and since the earlier diagnosis of MD in Nigeria [9–11], early reports records from Veterinary Hospitals and fieldworks indicated that there have been rapid and regular reported clinical case across the country by poultry farmers [12, 13].

Therefore, this study was designed to develop a protocol for a precise confirmatory diagnosis of Marek's disease by employing histopathology, immunohistochemistry, polymerase chain reaction (PCR) amplification of a fragment MDV gL gene and sequence analysis in Nigeria for optimized and enhanced early detection of lesions and management of Marek's Disease which is still a major problem in our poultry industry.

## 2. Materials and Methods

### 2.1. Sample Collection, Storage, and Gross Examination

Liver, proventriculus, intestine, spleen, and heart samples were collected at postmortem of two pullets. These organs were prioritized because of the nature of the infiltrative lesions (nodules).

They were examined grossly for nodular or neoplastic lesions. Appropriate sections of the liver, proventriculus, spleen, and heart were fixed in 10% neutral buffered formalin for histopathology, while liver samples were put in 30 μL virus transport medium in Eppendorf tubes and kept at −20°C until tested using PCR.

### 2.2. Histopathology

The formalin-fixed tissues were trimmed and routinely processed in an automatic histokinette machine [14]. The tissues were routinely embedded, and 4-5 μm tissue sections were made and stained for histopathological examination with the Haematoxylin and Eosin stain (H&E).

### 2.3. Immunohistochemistry

Immunohistochemistry was performed as previously described by Oyagbemi et al. [15]. Sections of 4 μm thickness were cut and floated from the paraffin-wax-embedded blocks on aminopropyl ethoxysilane-coated slides for immunohistochemical staining of MDV Meq antigen using the kit (Thermo Scientific, USA) and following standard procedures. Briefly, the slides were deparaffinized, and epitopes were retrieved by the heat-induced method. Endogenous peroxidase and receptors were blocked. The MDV antigens were detected using primary Marek's disease (1G6) monoclonal antibody (Santa Cruz Biotech, USA) diluted in TBST (1 : 50) according to manufacturer's instruction and applied to the slides and incubated overnight at 4°C. Biotinylated secondary antibody, Streptavidin–Biotin Complex (SABC) reagents, and 3,3 diaminobenzidine DAB as chromogen were added sequentially following washing steps with wash buffer. Similar tissues without antibody staining were also used as controls. The sections were counterstained with Meyer's haematoxylin and passed through alcohol and xylene before mounting. The slides were evaluated under a light microscope for reactivity.

### 2.4. DNA Extraction, PCR, and Sequencing

Total DNA was extracted from the liver samples using the DNeasy DNA extraction kit (Qiagen®) while amplification was done using the primers gL: forward primer: 5′- ATG AAA ATT TAT AGA GTA CTC GTG -3′ and reverse primer: 5′-GGC ATT GGC TCG TCG GCT-3′ following standard protocol; 94°C for 2 min for initial denaturation, 35 cycles of 95°C for 30 s, 50°C for 1 min, 72°C for 1.5 min, and final extension at 72°C for 10 min. PCR amplicons were purified using GeneJET PCR Purification Kit (ThermoSCIENTIFIC®, Pittsburgh, PA). Automated nucleotide sequencing was performed on an ABI 3130XL. Nucleotide sequences were viewed and edited with Chromas 2.6.6 (Technelysium, South Brisbane, Australia). The sequences were designated NGA_Ib_L1 and NGA_Ib_L2. These nucleotide sequences were compared with other MDV gL gene sequences in the GenBank database by means of a BLAST search conducted on the National Center of Biotechnology Information (NCBI) website (https://www.ncbi.nlm.nih.gov/BLAST/). Multiple sequence alignment of the sequences from this study and sequences retrieved from the GenBank was carried out using the BioEdit 7.0.5.3 computer software package using the Clustal W algorithm [16]. A maximum likelihood phylogenetic tree, with bootstrapping at 1000 replicates, was constructed using the MEGA XI computer software package [17]. Phylogenetic tree construction involved two sequences from this study and 18 avian alphaherpesviruses gL gene sequences retrieved from the GenBank. The sequences are the following: GHV2 HNLC503 (MG518371), GHV2 MD70/13 (MF431495), GHV2 LTS (KU744557), GHV2 Polen5 (MF431496), GHV2 EU-1 (MF431494), GHV2 ATE2539 (MF431493), GHV2 J-1 (KU744555), GHV2 RB-1B (EF523390), GHV2 Md5 (AF243438), GHV2 GA (U04994), GHV2 CVI988-Rispens (DQ530348), GHV2 648a (JQ836662), GHV3 (NC_002577), GHV3 HPRS24 (AB049735), MeHV1 (AF291866), MeHV1 (KT193609), GHV1 (MK895003), and GHV1 ILTV.157/19 (MK894999). Avian leukosis virus envelope (ALV env AY897231) gene was used as the out-group.

## 3. Results

### 3.1. Background History

Two carcasses were submitted for postmortem examination at the poultry diseases clinic, Department of Veterinary Medicine, University of Ibadan, on 22 October, 2018. The farmer complained of progressive loss of condition, emaciation, and death. Prior to presentation, farmer had lost a total of 58 birds in one week without obvious signs of paralysis. The birds were 22 weeks-old Bovans black and flock size was 2000 birds. Farmer purchased birds at day-old from a poultry hatchery who claimed that birds were vaccinated at hatching with CVI988/Rispens (attenuated Gallid alphaherpesvirus 2).

### 3.2. Pathology

**Figure 1:**
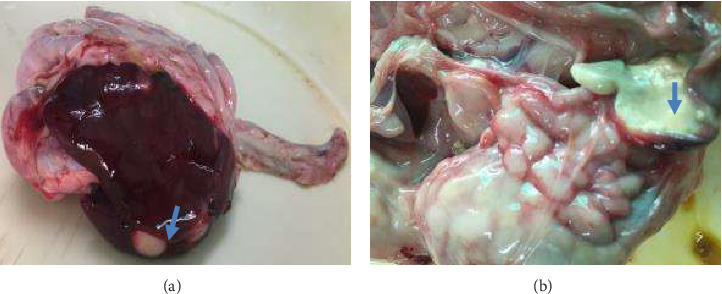
Photograph showing neoplastic nodules in (a) liver and (b) proventriculus (arrows).

**Figure 2:**
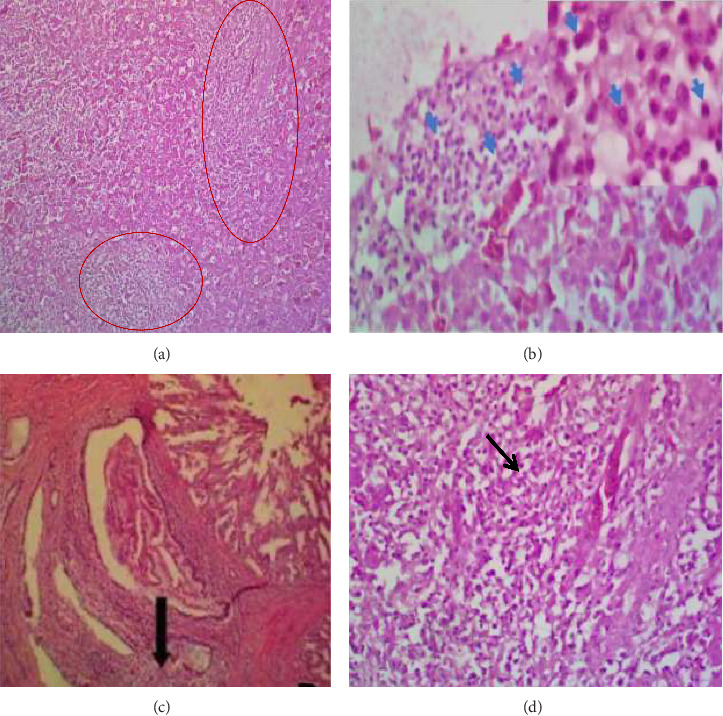
Photomicrographs showing foci of neoplastic cellular infiltrates. (a) Avian liver with multifocal areas of neoplastic cellular infiltration (circles). H&E × 160, (b) higher magnification of A H&E × 400. Inset showed morphological details of neoplastic cells (arrow). H&E × 1000. (c) Multifocal neoplastic cellular infiltration in the proventricular submucosa mucosa (arrow). H&E × 160. (d) Multifocal neoplastic cellular infiltration in the interstitium (arrow) of the heart. H&E × 400.

Gross lesions found included diffuse/nodular tumors in the liver, spleen, lung, proventriculus, intestine, and heart, particularly within the endocardium. The sciatic and brachial nerves were normal. The nodules were multiple and whitish (> 2 cm diameter) on the liver, proventriculus, intestine (Figures 1(a) and 1(b)), spleen, and heart. Microscopically, there were multifocal areas of neoplastic cellular infiltration consisting of lymphoblasts, lymphocytes, macrophages, and heterophils in the liver, heart, proventriculus, and intestine (Figure 2). The neoplastic cells were aggregating around blood vessels and compressing on adjacent parenchyma cells.

### 3.3. Immunohistochemistry

**Figure 3:**
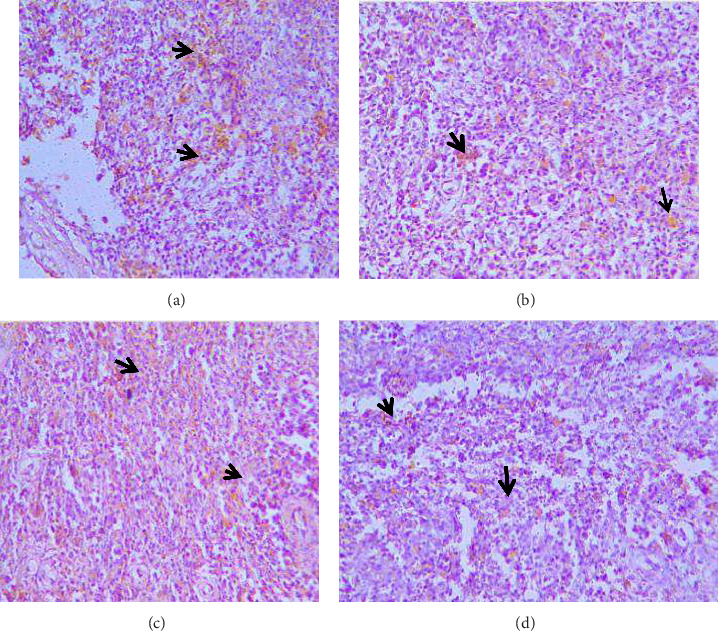
Immunohistochemical detection of MDV antigen (a) shows moderate positive staining of MDV antigens (arrows) within aggregates of neoplastic tumor cell in the spleen. (b) Similar MDV antigens (arrow) seen in the liver. (c) Intense positive MDV antigen (arrow) staining in heavily infiltrated heart tissue by neoplastic cells. (d) Mild positive MDV antigen (arrow) in neoplastic infiltrates within the submucosal glands of the proventriculus. X400.

The Marek's disease viral antigens were localized in the spleen, liver, and proventriculus (Figure 3).

### 3.4. PCR and Agarose Gel Electrophoresis

**Figure 4:**
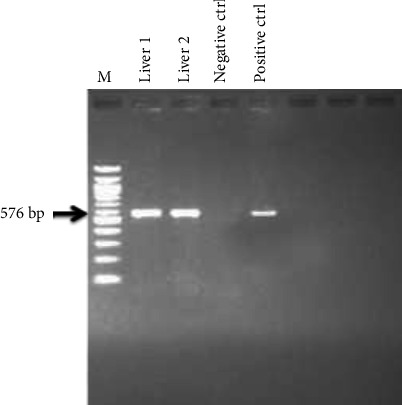
Agarose gel electrophoresis of 576 bp portion of gL gene of Marek's disease virus. Lane 1: Molecular marker, lanes 1-2: Liver samples, lane 3: negative control, and lane 4: positive control.

All the samples were positive with a molecular weight of 576 bp a fragment of MDV gL gene (Figure 4).

### 3.5. BLAST and Phylogenetic Analyses

**Figure 5:**
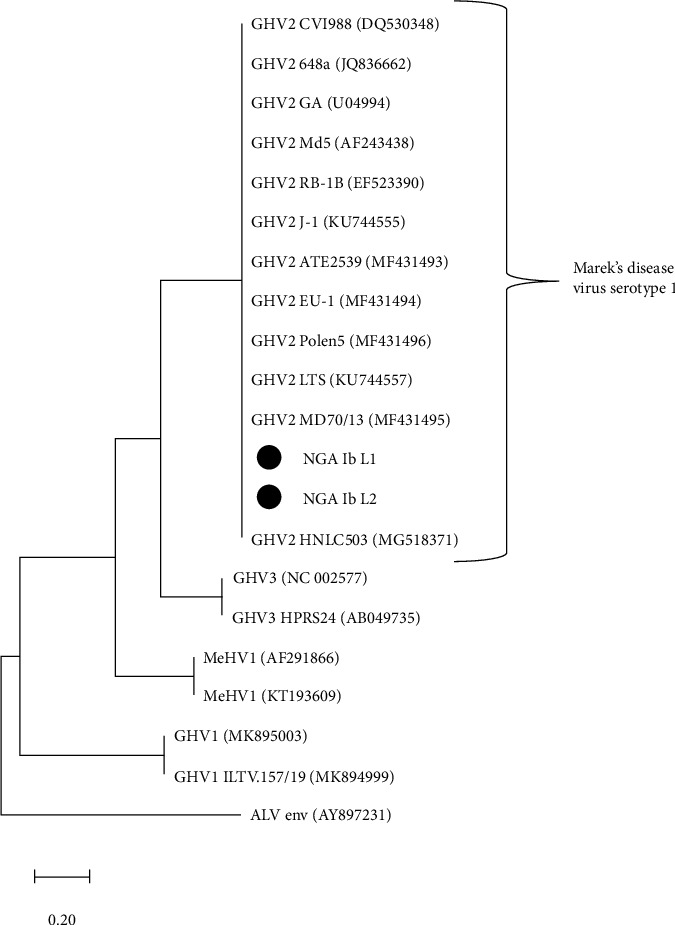
Phylogenetic analysis of Marek's disease virus serotype 1 and some other alphaherpesviruses based on gL gene nucleotide sequences. Phylogenetic tree was constructed via multiple alignments of 576 bp nucleotide sequence of gL gene from 18 alphaherpesviruses. Avian leukosis virus envelope gene was used as the out-group. The tree was analyzed by maximum likelihood method with bootstrapping (1000). Sequences from this study clustered with Gallid herpesvirus-2. Bar, 0.20 nucleotide substitutions per site.

BLAST analysis of MDV 1 sequences in the GenBank aligned with the sequences from this study was 100% similar including alignment with the vaccine strain sequences CVI988-Rispens (DQ530348). Sequences from this study clustered with Gallid herpesvirus-2 (MDV serotype 1), details shown in Figure 5.

## 4. Discussion

Marek's disease is still of significant concern in poultry farming in Nigeria. The incidence of Marek's disease despite vaccine coverage has been a major concern in the poultry industry as it confers great distress in flocks [18]. There has been an evolution of increasingly virulent strains of MDV, with concomitant suppressed immunity in chickens causing reduced productivity, cessation of hatchability, low performance rate, and mortality. The clinical signs of lymphoid leukosis are mostly nonspecific with inappetence, diarrhea, emaciation, and weakness. As such, Marek's disease may be confused with lymphoid leucosis but a distinguishing feature is that no paralysis is seen in the latter. However, the clinical signs of progressive loss of condition, emaciation, and death observed in the birds were typical of MD, though signs of paralysis now seem to be rare. This is now becoming a recurring feature on the field. It thus highly suggests the visceral form of MD. The contagious and lymphoproliferative nature of MDV has been accredited to a number of specific genes and their product. In this study, gross pathology, histopathology, immunohistochemistry, and molecular technique were used to diagnose Marek's disease from tissue samples derived from the carcass of commercial pullets suspected of having the disease.

Gross and histological lesions were evident in liver, proventricular, and heart tissues, showing variations of neoplastic cellular infiltration. The pleomorphic cell types are quite typical of MD. Considering the availability and proximity to research facilities like ours, there is a need to update clinicians on the current nature of MD and encourage them to do diagnostic work up on any possible lymphoproliferative case. The tools employed in this investigation are still very much relevant and specific in the diagnosis of lymphoproliferative conditions. PCR was used to detect the presence of gL genes which play a role in viral entry and cell infectivity in the expression of MDV in susceptible hosts. The gL gene was detected in the two liver samples with amplicon sizes of 576 bp that is in accordance with a study carried out by Mescolini et al. [19–21]. Phylogenetic analysis confirmed the causative agent to be MDV 1 proven by the clustering of the two sequences from this study with the MDV 1 sequences retrieved from the GenBank.

The 100% similarity of the GHV 2 (MDV 1) gL sequences from this study and most gL sequences from the GenBank including the sequence of the vaccine strain CVI988-Rispens (DQ530348), shows that the gL gene is relatively conserved and cannot differentiate the vaccine strains from wild-type MDV 1 or virulent MDV 1. However, with of immunohistochemistry technique in this study employing the monoclonal antibody 1G6 which detected MDV 1 Meq antigen/protein which is different structurally from the MDV vaccine LMeq protein, to the best of our knowledge, we detected the causative MDV 1 in the tissues, not the vaccine virus. This is a very reliable approach to precise diagnosis of Marek's disease in poultry.

## Data Availability

The data that support the findings of this study are available from the corresponding author upon reasonable request.
